# Vulcanite

**Published:** 1870-03

**Authors:** 


AMERICAN JOURNAL

OF

DENTAL SCIENCE.

Vol. III. THIRD SERIES--MARCH 1870. No. 11.

### ARTICLE I.

### *Vulcanite.*

By Professor Austen.

Within the last decade, dentistry has suffered its severest loss, through its greatest gain. Invention has given to the dentist no material more useful that vulcanite; none, the use of which has proved more injurious. This seeming contradiction, this *Dental Paradox*, is only one of the oft recurring instances of man's perverted ingenuity in converting benefits into injuries; proving, if proof were needed, the law of the moral world, that the worst of all curses is a blessing abused. We say "moral world," for whilst the utility of vulcanite is physical, the evil effects of its misuse are moral. The material is no more censurable for the harm done, than the dagger of the assassin is guilty of murder. Yet it is wise to take dangerous tools out of the reach of the unprincipled; wiser still, to place the actors themselves, where they can do no mischief; wisest of all, to instill such sense of moral obligation as makes restraint unnecessary.

The remarkable properties of "Hard Rubber," attracted the attention of certain dentists, more than fifteen years ago.

The very cumbrous apparatus, and the very tedious processes then thought necessary, interfered with a recognition of its merits. The few, who did recognize them, persevered in its use ; inventive genius simplified the details of the work, and results are now obtained, within reasonable time, by compact apparatus. The almost universal use of the vulcanite to day is a tribute to the sagacity of those, who, in 1858-60, withstood the storm of obloquy and abuse that assailed those who ventured to suggest that a material could, in any respect, be equal, or superior, to gold.

"Familiarity breeds contempt," is a social proverb, but it applies, with even more force, to the habitual non-appreciation of the qualities of things which we are daily using. The writer can never forget his first incredulity, when shown a hard rubber comb—his astonishment, when convinced that it was only another form of that pliant plastic thing, known as rubber. Many readers will recall similar emotions. The same property of vulcanite exists to day; nor is it the less wonderful, because its availability has been simplified, difficulties removed and anticipated objections found to have no foundation in fact.

"A substance so plastic that it can be readily moulded to the most complex shape; copying the minutest detail and retaining its form, whilst passing into the condition of a hard, strong, elastic, light and unchanging material."

This is the invaluable gift of some inventor (Goodyear, or whoever else he may be) to dentistry. Did he cast his pearl before swine and have they turned again to rend him ? So the patentees seem to think and are laboring hard to "ring the nose" of the profession. As this is one ground of the present disfavor of vulcanite, let us give it a little attention.

When the member of a profession patents any invention or discovery, yet prides himself upon the *liberality* of his calling, he is open to the charge of inconsistency. We leave him to "fight it out on that line," and cannot here discuss the point. His right to reap the reward of toil is indisputable ; whether he shall take pay in honor or in hard cash, is a matter of taste; he can seldom get both. But a patent once secured, its validity sustained—it is as much entitled to respect as any other species of property.

The sale of a patent-right involves mutual obligation. One party pays for the privilege of using; the other guarantees to protect against the competition of those, who attempt to use without purchase. The rubber patent is enforced by parties, who fail to make good this guarantee of protection. Hence many honorable dentists refuse further compliance with a compact which has been violated. Mutual recriminations, law suits, actual and threatened and a host of vexations arise. Patentees think dentists generally a pack of thieves and are in turn charged with being a set of swindlers.

Now, in fact, under both charges, lies some show of truth. Thieves abound everywhere. Some steal bread, some money, some brains—or rather brain work. Theft of money is an unpardonable sin; it is the world's idol and must be duly respected; necessity may pardon the theft of bread; but the theft of brains—identify your property and recover as best you can! Meanwhile the thief profits by your work and does not suffer in reputation. All professions are infested with thieves of the third class, and dentistry is not exempt. They are hard to catch, harder to punish. For the satisfaction of defrauded inventors, we will suggest a way to detect and if they see fit, to expose such. When you see a dentist loud in his abuse of patents, vociferous in his praise of generous liberality, whilst having a little corner in his office, or drawer in his instrument case, which he is slow to exhibit to you—set him down as a "brain thief" of first water. He will steal an idea from you to day and sell it the next week, to his student or best friend, for fifty dollars.

But the existence of such, though a reproach to any calling, cannot disprove the fact that the majority of the dental profession are willing to pay any reasonable price for the use of a valuable patent, provided always the patentee sustains them against infringement.

As regards the rubber patent; for years no one knew whom to pay—even now, many do not know. Whilst hundreds use it with impunity, many will not touch it, although they have a high opinion of its value; because they will neither incur the charge of using what they have no right to; nor submit to the imposition of paying those who are not entitled to the money. This patent, so far as dentistry is concerned, has been outrageously mismanaged. It is mainly due to this, that the proprietors thereof find so much trouble in enforcing their "rights." Perhaps the most fatal errors are—first, the attempt made to compel dentists to violate professional confidence and furnish lists of their patients. Secondly, the clause in licenses which requires the owner to become a public informer. A national tax is a fruitful source of lies, and *royalty charges* occasion many false statements. We should be very glad to see the dental profession exempt from such corrupting temptation.

The remedy, in our judgement, for these vexatious ills, complained of alike by buyer and seller of the vulcanite patents, is to retain the privilege of manufacturing the material and selling it at a *very high price.* It would greatly benefit both patentee and dentist, if the rubber used in every piece cost not less than *five* dollars. The patentee can control the manufacture of rubber more easily than he can its use, and *cheap dentistry* would receive a dangerous wound—we had almost said death blow, but this dragon has more heads than that slain by England's champion saint. He has fattened and grown strong on vulcanite and his devastations are proportionately increased. We greatly fear that his St. George is not yet born.

And this brings us to the true explanation of the mischief wrought by vulcanite. Not because it has failed to sustain the promise of usefulness, it first gave. Its merits are such that, for certain important uses, its place cannot be supplied by any other known material. Not because of the annoyances of the Goodyear patent agents; although these are serious enough and have led to much demoralization. But because dentist themselves have failed to recognize the true value of the material; because they have made an incidental quality its prominent advantage and their real motive for using it, namely its cheapness.

They have used it thus in that underbidding of rival practitioners, which is the burning disgrace of Dental practice. They have advertized their cheap wares to the world, till the community look upon "vulcanite" as a material which costs nothing, and think that the work spent on it has not much more value. Whereas, ten years ago, they could scarely overcome a patient's prejudice in favor of gold; now, they have so thoroughly demoralized the people, that they will not have gold when the necessity of the case requires it.

Were it merely a question of cheapnesss it would not be so serious; for it is a maxim of political economy, that he is a public benefactor, who reduces the cost of articles of necessity. But it is no benefaction, when the value is reduced in like proportion, and it becomes an imposture, when the value is still more reduced. Cheap dentistry is like "dollar stores" and "gift enterprises"—you may possibly get the value of your money—you most probably will not. Now and then, a dollar may buy ten dollars worth; but, in the long run, the inevitable laws of barter will prevail, and the dollar will buy only the dollar's worth.

There is no more certain law than that cheap work leads to bad work. If proof of this were needed in dentistry—look at the shapeless, inartistic, badly fitting lumps of rubber, stuck over with staring bits of porcelain, which issue from a thousand dental offices—*false teeth*, most appropriately so named—*false* to every idea of truth, beauty and fittness; made by men, *false* to all proper sense of professional pride and duty; worn by persons, *false* to their own self-respect and dignity, in permitting themselves to be thus disfigured and made ridiculous.

A dentist, who is mean enough to get work by underbiding, will be dishonest enough to make it correspond to the price. And thus it has come to be a common, and alas! an accepted excuse for not doing what the case requires— "I cannot afford it at the price I get." Hence cheap dentistry is dishonest dentistry, when the price is such as to prohibit the fullest exercise of skill.

A dentist who works at prices, which demand unceasing toil to earn his daily bread, leaves himself no time for mental culture. His work becomes a drudgery, in no respect more elevating than that of a common day labourer. Hence cheap dentistry is degrading dentistry.

We sum up our charge against vulcanite—or more correctly its abuse—in that its cheapness and a certain facility of construction (in its crudest forms) have tended greatly to foster Cheap Dentistry and so to make dental art slovenly, dishonest and degrading. Whilst its peculiar properties are calculated in a high degree to develop art and skill, it has been so used as to lessen the demand for both, and to encourage a community to prefer economy to artistic workmanship. For this reason we regret that when, ten years ago, we predicted its universal adoption, based on consideration of its value, our prophetic vision did not forsee its sad misuse. Its abusers *then* were its opponents, maintaining their case by many absurd and false statements; its abusers *now* are its adherents—alas! that they ever ceased their opposition. Let us hope that, when the days of greenback currency are numbered, cheap vulcanite work will also breathe its last, and both mercantile and dental world have new life infused into them by a return to the old fashioned Gold Basis.

---

